# All β Cells Contribute Equally to Islet Growth and Maintenance

**DOI:** 10.1371/journal.pbio.0050163

**Published:** 2007-05-29

**Authors:** Kristen Brennand, Danwei Huangfu, Doug Melton

**Affiliations:** 1 Department of Molecular and Cellular Biology, Harvard University, Cambridge, Massachusetts, United States of America; 2 Harvard Stem Cell Institute, Harvard University, Cambridge, Massachusetts, United States of America; 3 Howard Hughes Medical Institute, Harvard University, Cambridge, Massachusetts, United States of America; Baylor College of Medicine, United States of America

## Abstract

In healthy adult mice, the β cell population is not maintained by stem cells but instead by the replication of differentiated β cells. It is not known, however, whether all β cells contribute equally to growth and maintenance, as it may be that some cells replicate while others do not. Understanding precisely which cells are responsible for β cell replication will inform attempts to expand β cells in vitro, a potential source for cell replacement therapy to treat diabetes. Two experiments were performed to address this issue. First, the level of fluorescence generated by a pulse of histone 2B–green fluorescent protein (H2BGFP) expression was followed over time to determine how this marker is diluted with cell division; a uniform loss of label across the entire β cell population was observed. Second, clonal analysis of dividing β cells was completed; all clones were of comparable size. These results support the conclusion that the β cell pool is homogeneous with respect to replicative capacity and suggest that all β cells are candidates for in vitro expansion. Given similar observations in the hepatocyte population, we speculate that for tissues lacking an adult stem cell, they are replenished equally by replication of all differentiated cells.

## Author Summary

The β cells of the pancreas are responsible for insulin production and their destruction results in type I diabetes. β cell maintenance, growth, and regenerative repair is thought to occur predominately, if not exclusively, through the replication of existing β cells, not via an adult stem cell. It was previously unknown, however, whether all β cells divide at the same rate, or if multiple subpopulations of β cells exist, some highly replicative and others very slowly dividing, possibly postmitotic. We performed two types of experiments to determine whether all β cells are alike: label-retaining analysis and clonal analysis. Our results indicate that all β cells contribute equally to islet growth and maintenance.

## Abstract 3

The pancreatic β cell pool is found to be homogeneous with respect to replicative capacity. This suggests that all β cells are candidates for the in vitro expansion required for replacement therapies.

## Introduction

Stem cells are defined by an ability to self-renew and differentiate into a variety of cell types. Some adult organs, including the intestine, skin, blood, and parts of the brain, are maintained by stem cells [1–5]. In cases where the differentiated cells are postmitotic, such as erythrocytes and olfactory neurons, tissue turnover depends entirely on stem cell differentiation.

To explain the mechanism of β cell maintenance and regenerative repair, it has been hypothesized that renewal occurs via an adult stem cell residing in the pancreatic ducts [6], acini [7], islets [8,9], spleen [10], or bone marrow [11]. In contrast, Dor et al. found that pre-existing β cells, rather than stem cells, are the major source of new β cells in healthy and pancreatectomized mice [12]. Furthermore, the forced cell cycle arrest of β cells severely restricts postnatal β cell mass [13], indicating that non–β cells (such as putative adult stem cells) cannot maintain β cell mass. Together, these results demonstrate that β cell mass is predominately, if not exclusively, sustained through the replication of β cells.

It remains unclear whether all β cells contribute equally to growth and maintenance. Two possible models might explain the expansion of β cells. The β cell population may be heterogeneous, comprised of both highly replicative cells and very slowly dividing, possibly postmitotic, cells. This would be consistent with the hypothesis that a subpopulation of insulin-expressing cells may maintain the entire pool, perhaps as unipotent adult stem cells [14] or by reversible dedifferentiation to a replicative state [15]. Alternately, the β cell population may be homogeneous, with all β cells contributing equally to growth.

**Figure 1:**
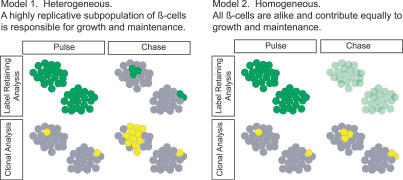
Two Possible Models for the Growth and Maintenance of Pancreatic β Cells Are Predicted, Given the Lack of an External Stem Cell Pool Two approaches were used to study replication of β cells. Pulse–chase analysis follows the loss of the H2BGFP label (green) with β cell division, while clonal analysis follows the generation of (yellow) clones from individual β cells. Model 1: the β cell population is heterogeneous, composed of fast- and slow-dividing subpopulations. This model predicts that highly replicative β cells will lose the H2BGFP label quickly and generate large clones, while slowly dividing β cells will fail to dilute the H2BGFP label and generate small clones. Model 2: the β cell population is homogeneous, and all β cells divide at the same rate. This model predicts that all β cells will lose the H2BGFP label uniformly, and that all clones identified through clonal analysis will be of comparable size.

Two approaches were used to address this issue (Figure 1). First, a broad survey of the replicative potential of the entire β cell pool was performed by monitoring the dilution or disappearance of a fluorescent marker accompanying cell division. β cells were pulse labeled with a tetracycline-inducible histone 2B–green fluorescent protein (H2BGFP) [16] and, following a chase period, the level of fluorescence detectable within β cells was measured. Second, the clonal descendents of individual β cells were examined using a reporter system developed for mosaic analysis with double markers (MADM) [17]. Both assays are designed to assess whether β cells are a heterogeneous population. If β cells are heterogeneous, highly replicative β cells will lose the H2BGFP label quickly as they replicate and generate large clones, while slowly dividing β cells will retain the H2BGFP label and generate small clones. Alternately, if β cells are a homogenous population, all β cells would be expected to lose the H2BGFP label at similar rates, and all clones should be of comparable size. We observed uniform loss of the H2BGFP label with time, and detected only similarly sized clones in the chase population. The tetracycline-inducible H2BGFP and MADM systems are complementary approaches, both supporting the conclusion that all β cells contribute equally to β cell growth and maintenance.

## Results

### H2BGFP Is Diluted with Cell Division In Vitro

**Figure 2:**
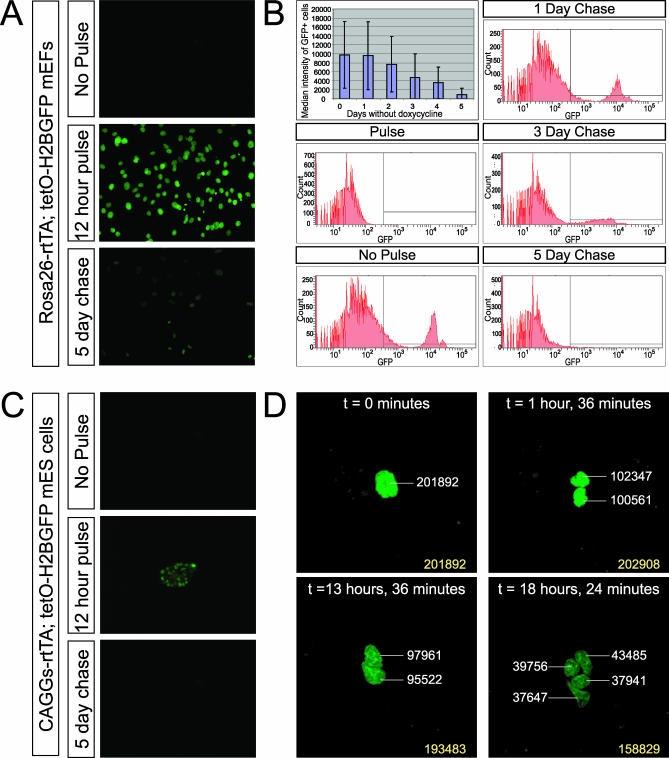
In Vitro Characterization of Dilution of Tetracycline-Inducible H2BGFP with Cell Division (A) Fluorescent images of Rosa26-rtTA; tetO-H2BGFP mEFs pulsed with doxycycline and cultured in the absence of doxycycline. Original magnification, 100×. (B) FACS plots of Rosa26-rtTA; tetO-H2BGFP mEFs pulsed with doxycycline and cultured in the absence of doxycycline. Median intensity of GFP-positive cells versus days cultured without doxycycline shown in graph. (C) Fluorescent images of CAGGs-rtTA; tetO-H2BGFP mES cells pulsed with doxycycline and cultured in the absence of doxycycline. Original magnification, 100×. (D) Time-lapse confocal images of a single CAGGs-rtTA; tetO-H2BGFP mES cell undergoing two rounds of cell division. Integrated pixel intensity shown in white text; total integrated pixel intensity of all cells shown in yellow text. Original magnification, 400×.

Tumbar et al. engineered transgenic mice expressing H2BGFP from a tetracycline-responsive promoter (tetracycline-inducible promoter [tetO]–H2BGFP) to mark cells and assess their rates of division [16]. To verify that H2BGFP is diluted with cell division and distributed equally between daughter cells, we characterized the tetO-H2BGFP system in vitro. Rosa26 and CAGGs (constitutive promoters containing the CMV enhancer and the chicken β-actin promoter) are commonly used in mouse embryonic fibroblasts (mEFs) and mouse embryonic stem (mES) cells. We used these promoters to drive expression of the reverse tetracycline transactivator (rtTA) in the presence of doxycycline. Rosa26-rtTA; tetO-H2BGFP mEFs and CAGGs-rtTA; tetO-H2BGFP mES cells express H2BGFP within 12 h of doxycycline application (Figure 2A and 2C). Doxycycline was removed from the media, and the progressive dilution of H2BGFP protein resulting from cell division was measured by fluorescence-activated cell sorter (FACS; Figure 2B and data not shown). A uniform loss of label was observed and the median GFP intensity of GFP-positive cells decreased with time. Given that mEFs divide every 24 h (unpublished data), and that H2BGFP fluorescence can no longer be detected after 5 d, H2BGFP fluorescence is no longer detectable above background in vitro by FACS after a population has undergone approximately five cell divisions (Figure 2B). The standard deviation of fluorescent intensity within the labeled pulse population is too large to precisely measure the number of cell divisions within the chase population.

To verify that H2BGFP is segregated equally between daughter cells, CAGGs-rtTA: tetO-H2BGFP mES cells were cultured on the stage of a confocal microscope and imaged every 12 min. After the first division, total GFP fluorescence in the two daughter cells, measured as integrated pixel inten-sity, added up to the fluorescence of the original cell, and H2BGFP was split equally between daughter cells in the first and second divisions (Figure 2D). In both dividing and nondividing cells, minimal bleaching was observed, despite imaging every 12 min over 18 h (Figure 2D and unpublished data). Detection of H2BGFP fluorescence is dependent on the laser settings used. In this case, H2BGFP fluorescence was no longer detectable after three rounds of cell replication, though lower laser intensities were used than typically employed for fixed tissue sections.

### H2BGFP Incorporation Is Replication Independent

Because β cells divide slowly, and some may be postmitotic, it was important to determine whether all cells, regardless of replicative activity, can be labeled by the inducible H2BGFP system. We found that H2BGFP labeling occurs independent of cell division in cultured Rosa26-rtTA; tetO-H2BGFP mEFs (Figure S1). Cells treated with mitomycin C are irreversibly arrested in S phase, but still express H2BGFP, which incorporates into the nucleus within 12 h of the administration of doxycycline. Furthermore, cells reversibly arrested with either aphidicolin (G_0_/G_1_ block) or nocadozole (G_2_/M block) express H2BGFP upon treatment with doxycycline. At 3 h after release from nocadozole, cells develop labeled mitotic spindles, indicating that the H2BGFP has been integrated into chromatin and not just added to a nuclear histone pool (unpublished data).

### In Postmitotic Cell Populations, H2BGFP Is Stable for at Least Six Months

**Figure 3:**
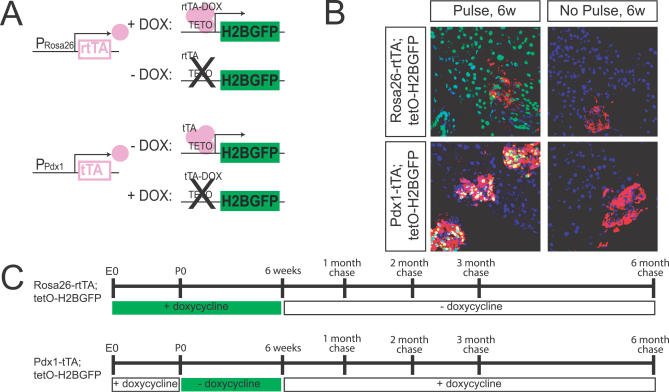
Experimental Schematic for Use of Tetracycline-Inducible H2BGFP To Identify LRCs In Vivo (A) Schematic for Rosa26-rtTA; tetO-H2BGFP and Pdx1-tTA; tetO-H2BGFP expression. (B) Doxycycline-dependent expression of Rosa26-rtTA; tetO-H2BGFP and Pdx1-tTA; tetO-H2BGFP expression in the pancreas. Insulin expression is shown in red. Up to 80% of ß cells are labeled following the pulse period; no ß cells are labeled in the absence of pulse. Original magnification, 400×. (C) Timeline for Rosa26-rtTA; tetO-H2BGFP and Pdx1-tTA; tetO-H2BGFP pulse–chase experiments.

**Figure 4:**
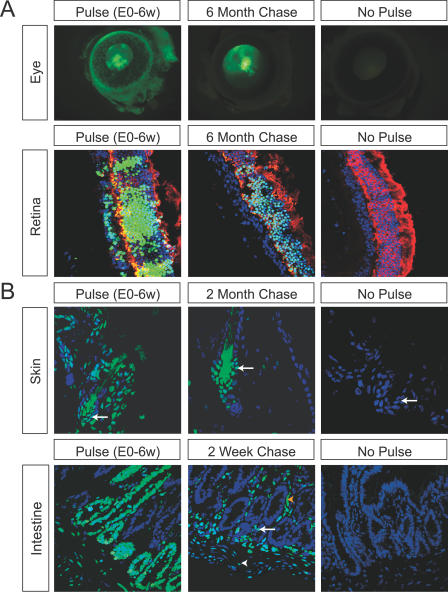
Identification of LRCs in Postmitotic Cells and Stem Cell Populations Indicates Stability of H2BGFP Fluorescence (A) LRCs detected in the postmitotic photoreceptor cells of the retina after expression of Rosa26-rtTA; tetO-H2BGFP from E0–E6 wk. Top row: H2BGFP fluorescence in the eye persists at least 6 mo following initial pulse. Original magnification, 20×. Bottom row: label retention in the retina is restricted to postmitotic photoreceptor cells, labeled in red with anti-recoverin. Original magnification, 400×. All images are exposure matched. (B) LRCs detected in the adult skin and intestine after expression of Rosa26-rtTA; tetO-H2BGFP from E0–E6 wk. In the skin, bulge stem cells are labeled with an arrow. In the intestine, a putative crypt cell is marked with an arrow. White arrowheads mark smooth muscle, and yellow arrowheads label enteric neurons. Images are exposure matched. Original magnification: skin, 630×; intestine, 400×.

The Rosa26 locus is active in most cells of the mouse [18], suggesting that in the presence of doxycycline, Rosa26-rtTA should drive tetO-H2BGFP expression and label most mouse cells. Rosa26-rtTA; tetO-H2BGFP labeled diverse cell types, including but not limited to pancreas, intestine, fat, bone marrow, muscle, skin, and retina (Figures 3, 4, and unpublished data), but not cortical neurons, olfactory bulb, or spinal cord, possibly due to the inability of doxycycline to cross the blood–brain barrier (unpublished data). Under repressive conditions (without doxycycline), no expression was observed in these organs (*n* = 4; Figures 3 and 4).

The stability of H2BGFP can be most easily assessed in postmitotic cells, where any loss of fluorescence with time can only be explained by degradation of the H2BGFP protein. Mammalian photoreceptor cells are postmitotic and are not replaced over the lifespan of the animal; they are identified by their position within the outer nuclear layer of the retina and by expression of the calcium-binding protein recoverin [19]. Photoreceptor cells can be labeled by Rosa26-rtTA; tetO-H2BGFP (Figure 4A). Following a chase of 6 mo (Figure 3C), H2BGFP was detected in whole eyes and sectioned retinas at the same imaging settings used to collect pulse data. Staining with recoverin verified that label retention is restricted to the photoreceptor layer of the retina (Figure 4A). Thus, the H2BGFP label is stable and retained in postmitotic cells. It is formally possible that the H2BGFP protein has a shorter half-life in pancreatic β cells than in postmitotic photoreceptor cells.

### LRCs Can Be Identified in Known Stem Cell Populations Using H2BGFP

As a further validation that the tetO-H2BGFP system can identify heterogeneity in cell populations, we confirmed that we could detect nonuniform loss of H2BGFP in tissues where slow-cycling cells are known to exist. Stem cells are often proliferatively quiescent compared with neighboring transit-amplifying cells. Because of their slow-dividing nature, stem cells tend to remain labeled in experiments that use DNA synthesis labels such as tritiated thymidine or bromodeoxyuridine. Label-retaining cells (LRCs) in Rosa26-rtTA; tetO-H2BGFP mice are clearly visible in the hair follicle bulge cells after a 2 mo chase, as previously shown by Tumbar et al. [16] (Figure 4B). Furthermore, GFP-positive intestinal crypt cells can be identified up to 1 mo following the pulse, though the vast majority of the labeled cells in chase intestines seem to be slowly dividing mesenchymal cells and intestinal neurons (Figure 4B). Finally, Lin^low^kit^+^sca^+^ sorted hematopoietic stem cells within the bone marrow also retain GFP fluorescence to a much greater extent than whole bone marrow (unpublished data). Because pulse–chase experiments using tetO-H2BGFP can identify tissue heterogeneity in the form of slowly dividing stem cell populations, it should also be able to identify a subpopulation of slowly dividing β cells, should it exist.

### No LRCs Are Detected in the β Cell Population

To determine whether all β cells divide at the same rate, we used the tetO-H2BGFP strategy in combination with promoters that express either tetracycline transactivator (tTA) or rtTA within the pancreas. Pdx1 expression in the postnatal pancreas is enriched in β cells, where it regulates insulin expression [20]. In tetO-H2BGFP animals, expression of tTA from the native Pdx1 locus (Pdx1-tTA) should label β cells in the absence of doxycycline, while the transgenic rat insulin promoter (RIP)–rtTA should label β cells in the presence of doxycycline. However, RIP-rtTA, but not Pdx1-tTA, labeled β cells even in unpulsed animals (unpublished data and Figure 3B), indicating that the RIP-rtTA transgene system is leaky. Therefore, Pdx1-tTA is the only β cell–specific driver suitable for these experiments. It should be noted that Pdx1-tTA animals are haploinsufficient; however, mice with one inactivated Pdx1 allele can be maintained in the heterozygous state and have normal pancreatic development and β cell maintenance, though they show modestly impaired glucose tolerance [20].

Pulsing Pdx1-tTA; tetO-H2BGFP animals for 6 wk after birth labeled 80% of β cells (Figure 3B). In addition to labeling β cells, some labeled nuclei occurred outside the β cell pool (Figure S2). Somatostatin and pancreatic polypeptide–expressing cells were frequently labeled in Pdx1-tTA; tetO-H2BGFP animals, consistent with the fact that Pdx1 was originally cloned from somatostatin-producing islet cell lines [21,22], and that Pdx1 expression can be detected in these cells [23]. Rare glucagon cells were labeled, while exocrine cells (amylase-positive) were consistently weakly labeled, and no H2BGFP expression was observed in the ducts (CK19-positive; Figure S2). These observations are also consistent with the findings of Oster et al., who observed rare Pdx1-immunoreactive nuclei in all pancreatic cell types [23].

**Figure 5:**
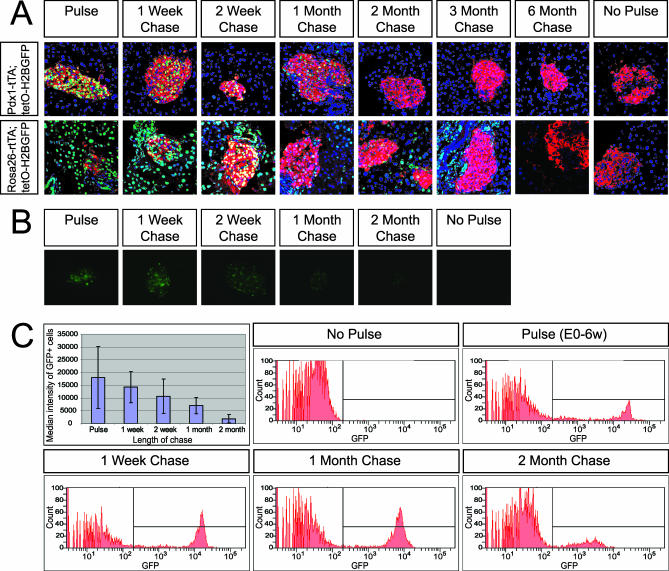
Uniform Loss of Label in Adult β Cells Following Pulse–Chase with Pdx1-tTA; tetO-H2BGFP and Rosa26-rtTA; tetO-H2BGFP Mice (A) Label retention present in the adult pancreas (insulin expression shown in red) following a chase period of up to 6 mo. Exposure-matched images. Original magnification, 400×. (B) Whole islets of Rosa26-rtTA; tetO-H2BGFP pulse–chase animals prior to dissociation and FACS. Exposure-matched images. Original magnification, 100×. (C) FACS plots of dissociated islets of Rosa26-rtTA; tetO-H2BGFP pulse–chase animals. Median intensity of GFP-positive cells versus length of chase shown in graph.

The entire β cell pool was assayed for LRCs. A group of 38 Pdx1-tTA; tetO-H2BGFP animals (19 female, 19 male) were pulsed from birth until 6 wk of age by ceasing doxycycline administration at postpartum day 0 (P0; Figure 3C). Six of these animals (three male, three female) were euthanized following a 6-wk postnatal pulse. All animals collected in the pulse group showed 80% of β cells labeled with H2BGFP; labeling was consistent throughout the pancreas and between animals (*n* = 6). The remaining mice were again administered doxycycline water to repress transcription of H2BGFP, and were euthanized after chase periods of 1 wk (*n* = 4), 2 wk (*n* = 4), 1 mo (*n* = 6), 2 mo (*n* = 6), 3 mo (*n* = 6), and 6 mo (*n* = 6). Sections of the pancreati were stained with insulin to identify β cells. We observed a uniform loss of label in β cells with time (Figure 5A).

To confirm this finding, the experiment was repeated with Rosa26-rtTA; tetO-H2BGFP animals. A group of 40 animals (18 female, 22 male) were pulsed from conception until 6 wk of age by administration of doxycycline water. Eight animals (four male, four female) were euthanized at 6 wk of age. Pulse expression in these mice was more variable between animals than for Pdx1-tTA; tetO-H2BGFP mice, so all experiments were performed on sibling cohorts. The remaining mice were removed from doxycycline and were euthanized after chase periods of 1 wk (*n* = 4), 2 wk (*n* = 4), 1 mo (*n* = 6), 2 mo (*n* = 6), 3 mo (*n* = 6), and 6 mo (*n* = 6). Sections of the pancreati were stained with insulin to identify β cells. Again, we observed uniform loss of label in β cells with time (Figure 5A).

To measure the relative intensity of GFP-positive cells, we used FACS analysis of dissociated islet cells. For these experiments, littermates were pulsed for at least 6 wk, and chases were structured so that all animals could be euthanized and analyzed by FACS on the same day. Importantly, comparisons of animals pulsed for 6 or 14 wk showed no significant increase in the median intensity of the GFP-positive population (unpublished data). By analyzing all timepoints in parallel, we were able to compare the relative GFP intensity between the pulse and chase populations. This experiment was repeated four times using Rosa26-rtTA; tetO-H2BGFP mice and twice using Pdx1-tTA; tetO-H2BGFP mice; the results were consistent each time. Exposure-matched photographs of whole islets taken prior to dissociation (Figure 5B) and FACS plots of dissociated islets (Figure 5C) indicate that the median intensity of the GFP fluorescence within the β cell pool decreases with time. No outlying population of LRCs in the β cell pool can be identified.

Hepatocytes, like β cells, are an endodermal cell type, and are thought to be maintained by self-replication [24]. It is unknown, however, whether all hepatocytes contribute equally to growth and maintenance. We examined livers from Rosa26-rtTA; tetO-H2BGFP pulse–chase animals and observed a uniform loss of label with time (Figure S3). Similar to the results with β cells, no outlying population of LRCs in the hepatocyte pool was identified.

### Clonal Analysis Demonstrates that β Cells Contribute Equally to Pancreas Expansion

**Figure 6:**
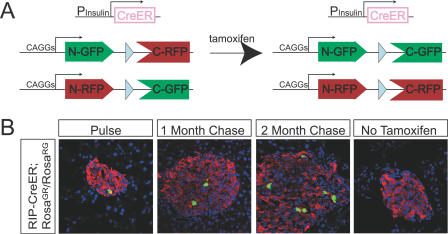
Clonal Analysis of β cells (A) Schematic for the RIP-CreER; Rosa26^GR^/Rosa26^RG^ pulse–chase experiments. (B) RIP-CreER; Rosa26^GR^/Rosa26^RG^ mice were pulsed with a tamoxifen injection between 4–8 wk of age. Shown here are sections of typical β cell clones (green) within 4 d of the pulse and after 1 and 2 mo of chase. β cells are identified by insulin staining (red). Original magnification, 400×.

To directly compare the replication capacity of individual β cells, a lineage-based clonal analysis in the pancreas was performed. RIP-CreER transgenic mice [12] drove expression of tamoxifen-dependent Cre recombinase specifically in β cells, while the MADM reporter system [17] was used to label individual β cells. The MADM system is a unique tool that allows low-frequency labeling of cells, a prerequisite for clonal analysis (Figure 6A). It contains two alleles at the Rosa26 locus, *Rosa26^GR^* and *Rosa26^RG^,* each containing reciprocal parts of chimeric marker genes (*GFP* and *RFP*) interrupted by a loxP site. Neither allele generates an active fluorescent protein until Cre-mediated interchromosomal recombination restores functional expression of *GFP* and *RFP.* Cells are labeled differently depending on when recombination occurs during the cell cycle. Recombination at G_0_ or G_1_ results in double-colored cells (expressing both *GFP* and *RFP*). Alternately, recombination at G_2_ results in two outcomes at equal frequency: either one red and one green daughter cell (single-colored cells), or one colorless and one double-colored daughter cell.

As expected, no labeling was observed in RIP-CreER; Rosa26^GR^/Rosa26^RG^ mice in the absence of tamoxifen. We injected tamoxifen over 3 d into 23 RIP-CreER; Rosa26^GR^/Rosa26^RG^ mice between 4 and 8 wk of age. Two animals were euthanized within 4 d of tamoxifen injection (the pulse group), and we found that 0.1%–0.5% of β cells were labeled. All clones observed were insulin positive, and 90% were single-cell clones (the remainder were composed of two cells).

In pulse and chase animals, all labeled cells expressed both *GFP* and *RFP* (*RFP* expression required antibody staining for detection; Figure S4A). The existence of only double-colored clones indicates that recombination occurred during G_1_ or G_0_, which is expected for a slow-dividing cell population that spends little time in G_2_. Conversely, in experiments performed using Pdx1-Cre; Rosa26^GR^/Rosa26^RG^ mice, where Cre recombinase was expressed in the rapidly dividing embryonic pancreas, single-colored clones were detected occasionally (Figure S4B). These observations are consistent with those of Zong et al. [17], who found that single-colored cells are a minority, though their proportion to double-colored cells increases when Cre recombinase is expressed in a rapidly dividing population. Given that all β cell clones are double-colored, all clones are shown only in green to allow for ease of presentation, and insulin staining is shown in red.

For the purpose of this experiment, clones are defined as clusters of labeled cells within a single islet; all cells within a clone are assumed to be derived from a single β cell. Tamoxifen-treated mice were euthanized 1 mo (*n* = 12) or 2 mo (*n* = 9) following the pulse. A total of 175 clones from 1-mo chase animals and 122 clones from 2-mo chase animals were sampled using single random sections, and no large clones were detected. The absence of large clones qualitatively suggests an absence of fast-dividing β cells. To estimate the expansion of β cells, we analyzed serial sections. After 1 mo of chase, the average size of a clone was 5.1 ± 5.4 cells (*n* = 45 clones), whereas after 2 mo of chase, the average size of a clone was 8.2 ± 6.9 cells (*n* = 40 clones). In addition, the experiment was repeated using CAGGs-CreER; Rosa26^GR^/Rosa26^RG^ mice, and again, no large β cell clones were detected (unpublished data). Labeled cells found within a single islet are of clonal origin: the probability that three or more labeled cells found within the same islet are not clonal is 1.8 × 10^−2^ (based on Poisson distribution analysis assuming labeling frequency of 0.1% and islet size of 500–1,000 cells). This clonal analysis supports the model that the growth and maintenance of β cell mass in the adult pancreas is achieved by the replication of individual β cells that have similar replicative capacities.

## Discussion

Our experiments were designed to address the mechanism of growth and maintenance of mature β cells. To determine whether all β cells divide at the same rate in the adult mouse, two experiments were undertaken. The tetracycline-inducible H2BGFP and MADM systems are complementary approaches: whereas tetO-H2BGFP labels most β cells and provides a broad view of the population dynamic, MADM labels single cells and provides an accurate clonal analysis of the progeny of individual cells within the β cell pool. Both the uniform loss of the H2BGFP label with time in the β cell population and the comparable β cell clone sizes generated through MADM analysis indicate homogeneity exists within the β cell pool. Stated otherwise, all β cells appear to contribute equally to growth and maintenance.

The β cell mass is dynamic and can respond to environmental cues such insulin and glucose [25]. The β cell number increases dramatically in the first year of rodent life [12,26], up to 10-fold in cases of insulin resistance [27], and up to 1.5-fold during pregnancy [28,29]. Recent experiments suggest that when not hindered by persistent autoimmune attack or the toxicity of high blood glucose levels [30], β cells have the capacity to regenerate. While the mechanism regulating β cell expansion remains unclear, our findings indicate that all β cells are capable of replication and are therefore viable targets for in vitro or in vivo expansion.

Seaberg et al. recently reported that single-cell clones derived from adult islets generated colonies of 2,000–10,000 cells that expressed markers of neural, glial, pancreatic endocrine, exocrine, and duct identities [31]. These clones were generated from ~0.02% of islet cells, though their identity and relationship to in vivo growth is yet to be determined. We cannot rule out the possibility that a rare type of β cell was missed in our examination of individual clones using the MADM marking experiments. However, because the rate of clonal expansion is sufficient to account for the growth of the β cell population during the chase period, a rare highly proliferative β cell did not contribute significantly to the expansion of β cell mass.

Published rates for β cell replication in adult mice (12 wk old) are highly variable, from 2% [32] to 15% per day [33]. Assuming 5% of β cells replicate per day, and that all β cells are equivalent, β cells should divide approximately every 20 d. This would dilute the H2BGFP label beyond detection (by completing up to five rounds of replication) within 100 d. In addition, clone size at 2 mo should be approximately eight cells. These straightforward calculations predict results that are entirely consistent with our findings. These estimates, of course, assume no β cell death over the duration of our experiments.

β cells have a finite lifespan, but the absolute β cell death rate is unknown. Based on β cell mass measurements and an estimate of β cell proliferation of 2% per day throughout adulthood, Finegood et al. calculated the β cell lifespan to be 52 d [32]. Recent findings demonstrate that β cell proliferation rates decline to less than 0.1% in 1-y-old mice [33], casting doubt on the often quoted rates for β cell turnover in mice. Furthermore, TUNEL analysis of wild-type β cells consistently fail to identify apoptotic cells [13,27,33,34]. Regardless of the true rate of β cell turnover, our findings of a uniform loss of label and a consistent clone size indicate that all β cells have equivalent replicative capacity.

Pancreatic β cells are not the only differentiated cell type capable of growth and maintenance without the support of an adult stem cell population. Hepatocytes are highly replicative and not thought to be supported by a facultative stem cell under normal conditions [35]. Pulse–chase analysis with the tetracycline-inducible H2BGFP label shows that all hepatocytes lose their label at the same rate. Therefore, like the β cell population, the hepatocyte population seems to be homogeneous. We do not know of an example of a mature differentiated cell type that has two populations (one replicative and the other not). We speculate that when tissues are without an adult stem cell, they are replenished by equal replication of all differentiated cells.

The demonstration that all β cells are equivalent, contributing equally to the growth and maintenance of the β cell population, has clinical implications if we assume that rodents and man use the same mechanism for pancreatic homeostasis. The destruction of β cells that causes type I diabetes has been counteracted by the transplantation of β cells. The clinical impact of this approach is currently limited, in part, by the scarcity of available pancreatic tissue [36]. A better understanding of adult β cell replication may help attempts to expand pancreatic β cells in vitro as a source of transplant material to treat diabetes.

## Materials and Methods

### Mice.

Pdx1-tTA, Rosa26-rtTA, tetO-H2BGFP, and MADM mice were generously provided by Ray MacDonald (University of Texas Southwestern Medical Center, Dallas, Texas, United States), Rudolf Jaenisch (Massachusetts Institute of Technology, Cambridge, Massachusetts, United States), Elaine Fuchs (Howard Hughes Medical Institute and Rockefeller University, New York, New York, United States), and Liqun Luo (Howard Hughes Medical Institute and Stanford University, Stanford, California, United States), respectively. Mice were maintained at a barrier facility in the Department of Molecular and Cellular Biology at Harvard University under animal protocol 93–15. Pdx1-tTA, Rosa26-rtTA, and tetO-H2BGFP mice were backcrossed to >95% C57BL/6 inbred background; RIP-CreER and MADM mice were maintained on a mixed background.

Greater variation of H2BGFP expression was observed in Rosa26-rtTA; tetO-H2BGFP animals than with the other drivers; experiments were conducted on littermates to reduce variability. Notably, the highest-expressing animals were often runted, indicating that either Rosa26-rtTA activity or H2BGFP expression in some cell types is harmful. Breeding to a C57BL/6 inbred background reduced variability to some extent.

### Genotyping.

Genotyping was performed by adding a tail biopsy to 100 μl DirectPCR (ViaGen, http://www.viagen.com) with 30 μg proteinase K (Roche, http://www.roche.com), incubating overnight at 55 °C and denaturing proteinase K for 20 min at 95 °C. PCR primers specific to tTA (forward 5′-ctggtcgagctggacggcgacgta aac-3′, reverse 5′-atgtgatcgcgcttctcgttgggg-3′), Rosa26-rtTA (A 5′-aaagtcgctctgagttgtTAt-3′, B 5′-gcgaagagtttgtcctcaacc-3′, C 5′-ggagcgggagaaatggatatg-3′), GFP (forward 5′-ctggtcgagctggacggcgacgtaaac-3′, reverse 5′-atgtgatcgcgcttctcgttgggg-3′), Cre (forward 5′-tgccacgaccaagtgacagc-3′, reverse 5′-ccaggttacggatatagttcatg-3′), MADM wild-type (forward 5′-ctctgctgcctcctggcttct-3′, reverse 5′-cgaggcggatcacaagcaata-3′), and MADM knockin alleles (forward 5′-ctctgctgcctcctggcttct-3′, reverse 5′-tcaatgggcgggggtcgtt-3′) amplified 600 bp, 300 bp, 600 bp, 600 bp, 330 bp, and 250 bp fragments, respectively. PCR conditions: 95 °C for 5 min, then 35 cycles of 95 °C for 30 sec, 55 °C for 30 sec, 72 °C for 60 sec, and finally 72 °C for 5 min.

### Doxycycline and tamoxifen.

Doxycycline (Sigma, http://www.sigmaaldrich.com) was added to drinking water at 1 mg/ml and sweetened with sucrose (1%). Water bottles were changed weekly with freshly prepared solution. Tamoxifen (Sigma) was dissolved in corn oil at 20 mg/ml and mice were injected intraperitoneally (6 or 8 mg/d for 3 consecutive days).

### Immunohistochemistry.

Tissue was dissected from mice, fixed in 4% paraformaldehyde/PBS solution for two hours at 4 °C, washed in PBS, incubated in 30% sucrose/PBS solution overnight, embedded in OCT (Tissue-Tek; Electron Microscopy Sciences, http://www.emsdiasum.com) and stored at −80 °C. Frozen samples were sectioned at 10 μm for staining or up to 50 μm for serial analysis. The following primary antibodies and dilutions were used: guinea pig anti-Pdx1 antibody (kindly provided by C. Wright, Vanderbilt University, Nashville, Tennessee, United States), 1:1000; guinea pig anti-swine insulin (DakoCytomation, http://www.dako.com), 1:200; guinea pig anti-glucagon antibody (Linco, http://www.linco.com), 1:200; rabbit anti-human pancreatic polypeptide (DakoCytomation) 1:200; rabbit anti-human somatostatin (DakoCytomation), 1:200; rabbit anti-amylase (Sigma), 1:200; rabbit anti-CK19 (Developmental Studies Hybridoma Bank, http://dshb.biology.uiowa.edu), 1:1,000; rabbit anti-GFP (Molecular Probes, http://probes.invitrogen.com),1:200; rabbit anti-DsRed (Clontech, http://www.clontech.com), 1:100; and rabbit anti-recoverin (Chemicon, http://www.chemicon.com), 1:2,000. Secondary antibodies donkey rhodamine RedX anti–guinea pig (Jackson ImmunoResearch, http://www.jacksonimmuno.com), donkey rhodamine RedX anti-rabbit (Jackson ImmunoResearch), and donkey rhodamine RedX anti-goat (Jackson ImmunoResearch) were used at 1:200 dilution. To visualize nuclei, slides were stained with 0.5 μg/ml DAPI and then mounted with VectaShield Mounting Medium (Vector Laboratories, http://www.vectorlabs.com). Triple-labeled GFP/rhodamine/DAPI images were acquired using a Zeiss LSM510 Meta confocal microscope (http://www.zeiss.com).

### Islet dissociation and FACS.

The pancreas was perfused through the bile duct with 5 ml digestion solution (low-glucose DMEM [Gibco, http://www.invitrogen.com] with 10 mM HEPES [Gibco], 0.25 mg/ml liberase RI [Roche], and 0.1 mg/ml ovalbumin trypsin inhibitor [Roche]), dissected and incubated at 37 °C for 20 min. Cold washing solution (low-glucose DMEM with 10 mM HEPES, 10% FBS [Hyclone, www.hyclone.com], and 0.1 mg/ml ovalbumin trypsin inhibitor) was added, and islets were centrifuged, washed twice, and filtered through a 500 μm diameter wire mesh. Islets were centrifuged, washed twice in washing solution, resuspended in Histopaque 1077 (Sigma), and vortexed. The islet suspension was carefully overlaid with washing solution (without serum) and centrifuged for 20 min at 10 °C, separating islets from exocrine tissue. The islet layer was collected at the interface, pelleted, washed twice, and further purified by two rounds of gravity sedimentation. Finally, pure islets were handpicked under a dissecting scope. Islets were dissociated by incubation with 0.25% trypsin-EDTA (Gibco) at 37 °C for 5 min, washed, fixed for 15 min in 1% paraformaldehyde/PBS solution, resuspended in 5% donkey serum/PBS, and FACS sorted on a BD Aria (BD Biosciences, http://www.bdbiosciences.com).

### Tissue culture.

Rosa26-rtTA; tetO-H2BGFP mEFs were obtained by collecting timed plugs and dissecting embryonic day (E) 12.5 embryos. Embryos were eviscerated, trypsinized, plated on gelatinized plates, and cultured in standard mEF media (DMEM with 10% FBS and 1x penicillin/streptomycin [Gibco]). Cells were grown with 10 μg/ml doxycycline to induce H2BGFP transcription.

CAGGs-rtTA; tetO-H2BGFP mES cells were obtained by electroporating a CAGGs-rtTA-IRES-puromycin plasmid into tetO-H2BGFP mES cells derived from blastocysts, selecting with 4 μg/ml puromycin in 10 μg/ml doxycycline and picking and expanding green colonies. mES cells were grown in standard mES conditions (knockout DMEM [Gibco] with 15% defined FBS [Hyclone], 200 mM L-glutamine [Gibco], 10 mM nonessential amino acids [Gibco],1× penicillin/streptomycin, 0.001% β-mercaptoethanol [Sigma], and 1,000 U/ml LIF [Chemicon]).

Dividing cells were imaged by culturing on the stage of a Zeiss LSM confocal microscope, and 15-image z-stacks were scanned every 12 min at 0.5% laser intensity. Images were quantified using MetaMorph software (Molecular Devices, http://www.moleculardevices.com), and values were recorded as integrated pixel intensity.

Cell-cycle experiments were performed as follows: 10 μg/ml mitomycin C (Sigma) was applied to cells for 3 h to irreversibly inhibit the cell cycle in S phase, 0.25 ng/ml aphidicolin (Sigma) or 100 ng/ml nocadozole (Sigma) was applied to cells for 6 h to reversibly arrest the cell cycle at G_0_/G_1_ or G_2_/M, respectively.

## Supporting Information

Figure S1 Labeling of Chromosomes with H2BGFP is Cell Replication Independent Rosa26-rtTA; tetO-H2BGFP mEFs express H2BGFP within 12 h of administration of doxycycline, even following cell-cycle inhibition. Cells treated with mitomycin C for 3 h are irreversibly blocked in S phase but still develop green nuclei within 12 h of doxycycline treatment. Administration of aphidicolin (G_0_/G_1_ block) or nocodazole (G_2_/M block) for 6 h prior to and during doxycycline treatment still results in labeled nuclei within 12 h. Original magnification, 200×.
(4.4 MB PDF)

Figure S2 Immunohistochemical Characterization of Pancreatic Marker Expression within the Pdx1-tTA; tetO-H2BGFP Pulse Population Red staining represents common pancreatic proteins: insulin expression in ß cells; glucagon staining, α cells; somatostatin, δ cells, pancreatic polypeptide, pancreatic polypeptide cells; amylase, exocrine cells; and CK19, duct cells. Top panel: original magnification, 400×. Dashed box represents area of magnification in bottom panel.
(6.1 MB PDF)

Figure S3 Uniform Loss of Label in Adult Hepatocytes Following Pulse–Chase with Rosa26-rtTA; tetO-H2BGFP Mice No label retention present in hepatocytes following a chase period of 1 mo. Exposure matched images. Original magnification, 400×.
(1.7 MB PDF)

Figure S4 *GFP* and *RFP* Expression in MADM-Generated Clones (A) Immunohistochemical staining showing expression of both *GFP* (green) and *RFP* (red) in a RIP-CreER; Rosa26^GR^/Rosa26^RG^ 2-mo chase β cell clone. Original magnification, 200×.
(B) Demonstration of single-colored red and green clones in Pdx1-Cre Rosa26^GR^/Rosa26^RG^ mice. Original magnification, 400×.
(5.8 MB PDF)

## Abbreviations

CAGG: a constitutive promoter containing the CMV enhancer and the chicken β-actin promoter
FACS: fluorescence-activated cell sorter
H2BGFP: histone 2B–green fluorescent protein
LRC: label-retaining cell
MADM: mosaic analysis with double markers
mEF: mouse embryonic fibroblast
mES cell: mouse embryonic stem cell
RIP: rat insulin promoter
rtTA: reverse tetracycline transactivator
tetO: tetracycline-inducible promoter
tTA: tetracycline transactivator
