# Correction: All ß Cells Contribute Equally to Islet Growth and Maintenance

**DOI:** 10.1371/journal.pbio.0050240

**Published:** 2007-09-18

**Authors:** Kristen Brennand, Danwei Huangfu, Douglas Melton

Correction for:

Brennand K, Huangfu D, Melton D (2007) All β cells contribute equally to islet growth and maintenance. PLoS Biol 5(7): e163. doi:10.1371/journal.pbio.0050163

The labels in Figure 2B indicating the “Pulse” and “No Pulse” FACS conditions for the Rosa26-rtTA;tetO-H2BGFP cells were switched. Labeled properly, the middle left panel should be titled “No Pulse,” and the bottom left panel should be titled “Pulse.”

