# Supplementary material for: All β Cells Contribute Equally to Islet Growth and Maintenance
Source: PLoS Biol. 2007 May 29;5(7):e163. doi: 10.1371/journal.pbio.0050163 (PMC1877817; doi:10.1371/journal.pbio.0050163)
Supplement: Supplementary file 2 [file pbio.0050163.sg002.pdf]

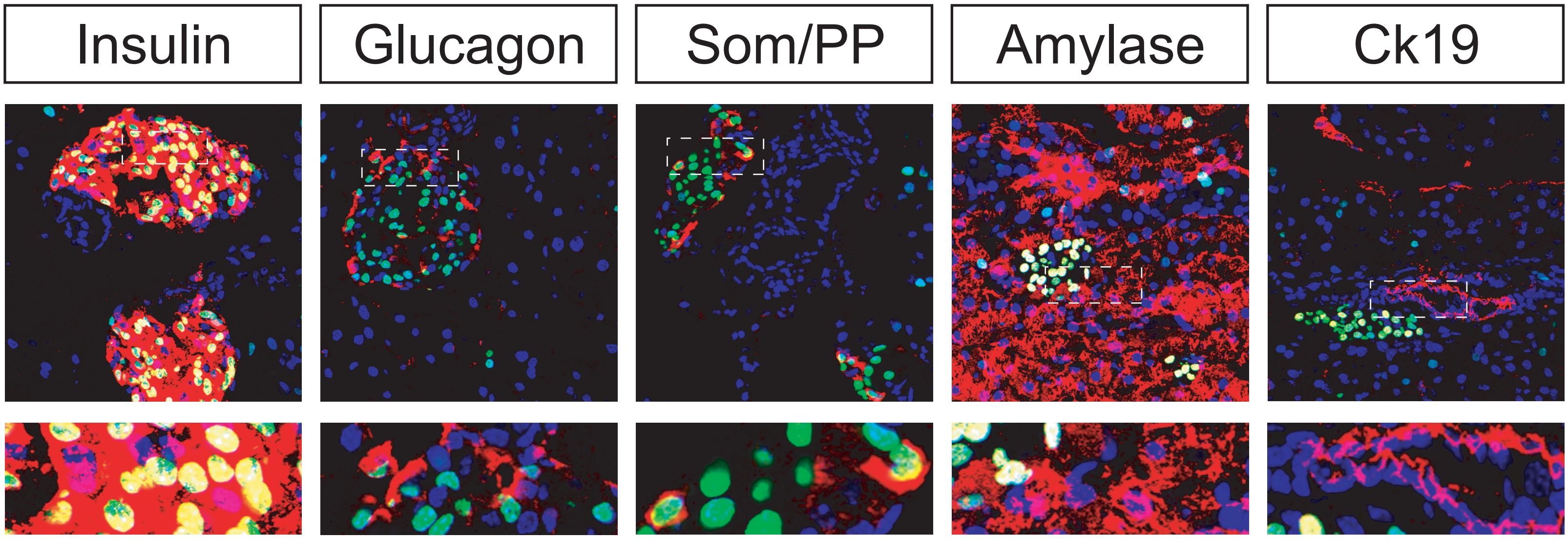
Insulin
Glucagon
Som/PP
Amylase
Ck19
