# Supplementary material for: All β Cells Contribute Equally to Islet Growth and Maintenance
Source: PLoS Biol. 2007 May 29;5(7):e163. doi: 10.1371/journal.pbio.0050163 (PMC1877817; doi:10.1371/journal.pbio.0050163)
Supplement: Supplementary file 4 [file pbio.0050163.sg004.pdf]

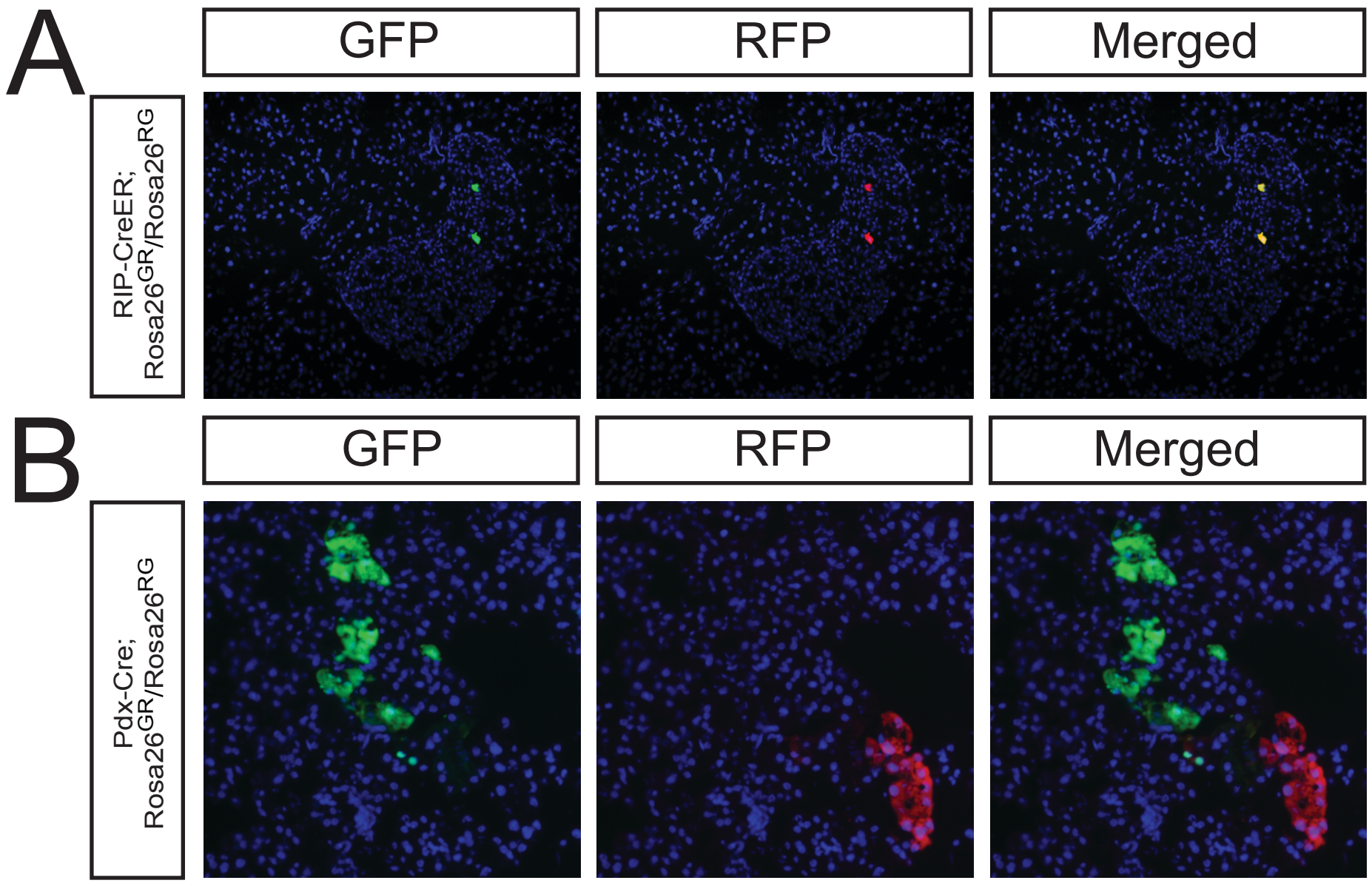
A
GFP
RFP
Merged
RIP-CreER;
Rosa26GR/Rosa26RG
B
GFP
RFP
Merged
Pdx-Cre;
Rosa26GR/Rosa26RG
